# Supplementary material for: Development of SLAF-Sequence and Multiplex SNaPshot Panels for Population Genetic Diversity Analysis and Construction of DNA Fingerprints for Sugarcane
Source: Genes (Basel). 2022 Aug 19;13(8):1477. doi: 10.3390/genes13081477 (PMC9408448; doi:10.3390/genes13081477)
Supplement: Supplementary file 1 [file genes-13-01477-s001.zip › Supplementary Figure S2.pdf]

Supplementary Figure S2. Histogram of genetic distance matrix and Cluster analysis diagram based respectively on 32 core SNP markers.

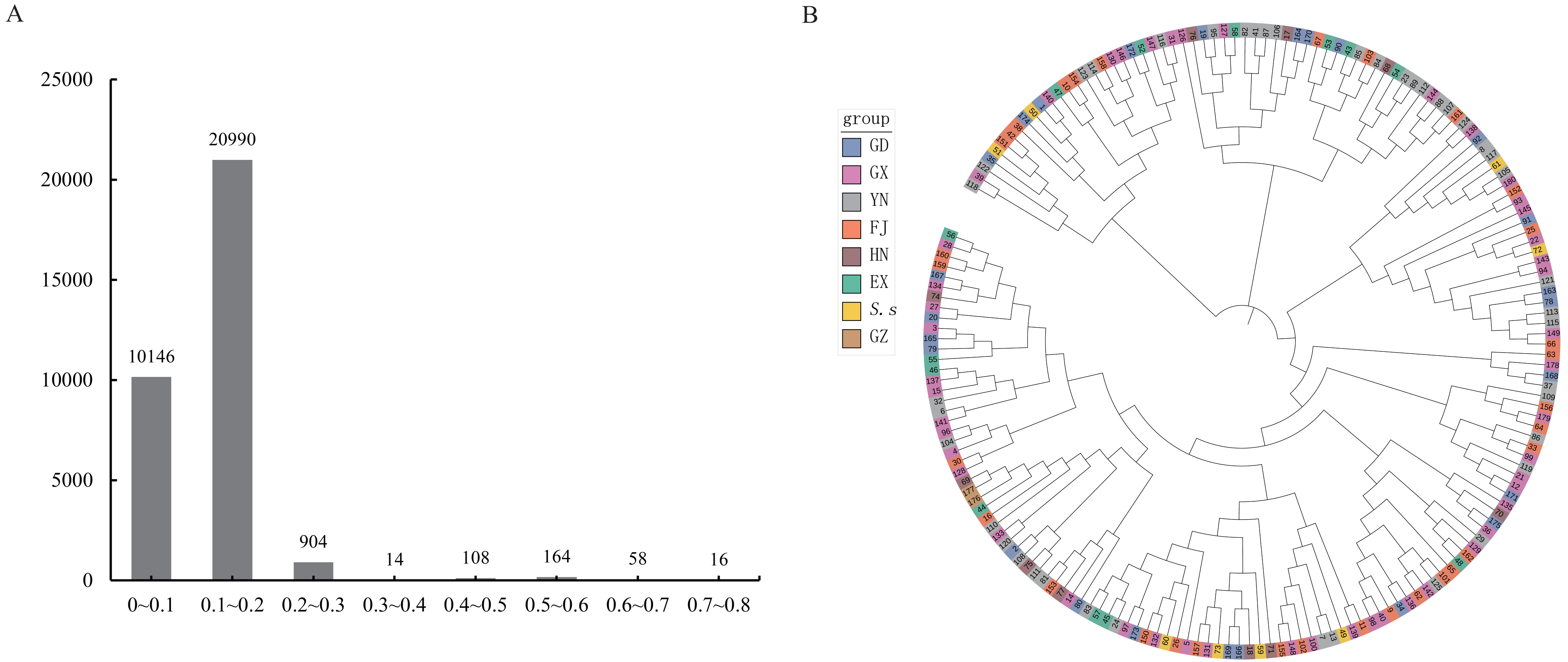

（A） Histogram of genetic distance matrix based on 32 core SNP markers. (B) Cluster analysis diagram based respectively on 32 core SNP markers.
