# Supplementary figures and images for: Development of SLAF-Sequence and Multiplex SNaPshot Panels for Population Genetic Diversity Analysis and Construction of DNA Fingerprints for Sugarcane
Source: Genes (Basel). 2022 Aug 19;13(8):1477. doi: 10.3390/genes13081477 (PMC9408448; doi:10.3390/genes13081477)

## Supplementary Figure S1

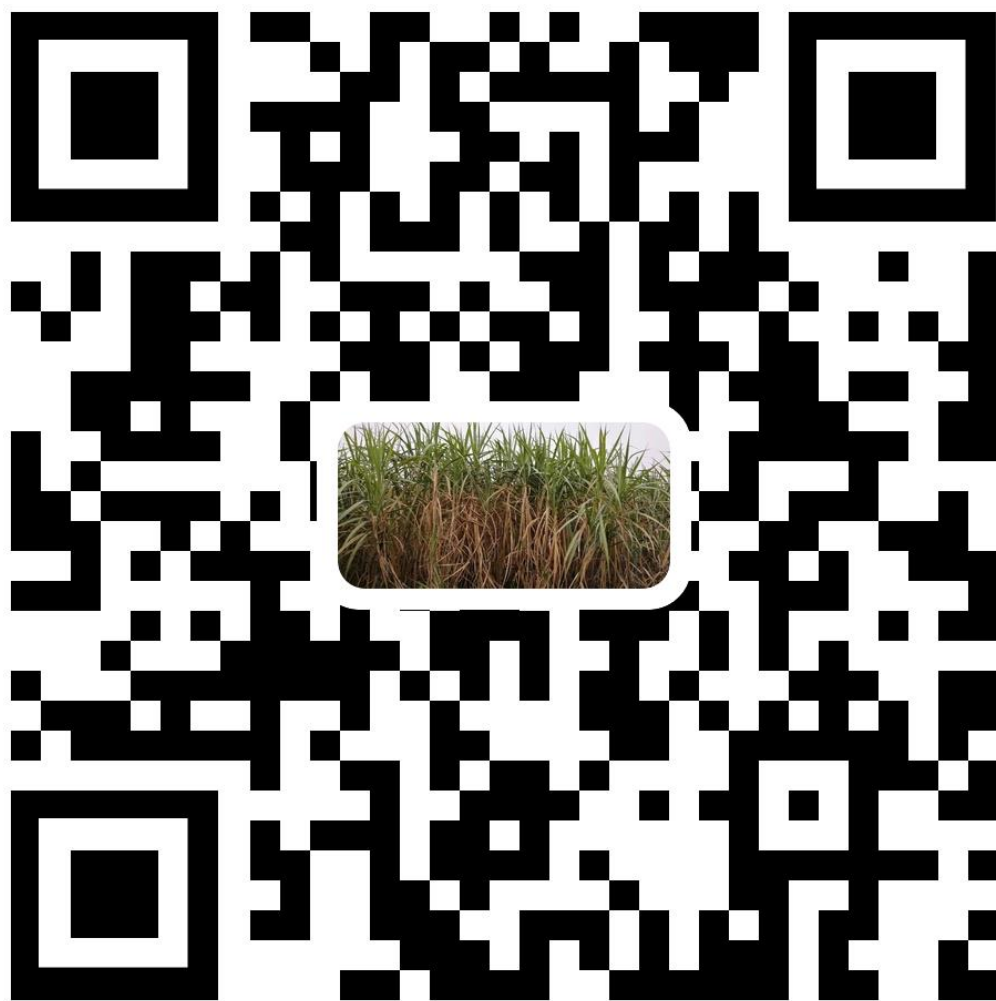

The QR code of Funong 41

Supplement: Supplementary file 1 [file genes-13-01477-s001.zip › Supplementary Figure S1 .pdf]
